# Reorganisation of primary care for older adults during COVID-19: a cross-sectional database study in the UK

**DOI:** 10.3399/bjgp20X710933

**Published:** 2020-07-14

**Authors:** Mark Joy, Dylan McGagh, Nicholas Jones, Harshana Liyanage, Julian Sherlock, Vaishnavi Parimalanathan, Oluwafunmi Akinyemi, Jeremy van Vlymen, Gary Howsam, Martin Marshall, FD Richard Hobbs, Simon de Lusignan

**Affiliations:** Nuffield Department of Primary Care Health Sciences, University of Oxford, Oxford.; Nuffield Department of Primary Care Health Sciences, University of Oxford, Oxford.; Nuffield Department of Primary Care Health Sciences, University of Oxford, Oxford.; Nuffield Department of Primary Care Health Sciences, University of Oxford, Oxford.; Nuffield Department of Primary Care Health Sciences, University of Oxford, Oxford.; Nuffield Department of Primary Care Health Sciences, University of Oxford, Oxford.; Nuffield Department of Primary Care Health Sciences, University of Oxford, Oxford.; Nuffield Department of Primary Care Health Sciences, University of Oxford, Oxford.; Royal College of General Practitioners, London.; Royal College of General Practitioners, London.; Nuffield Department of Primary Care Health Sciences, University of Oxford, Oxford.; Nuffield Department of Primary Care Health Sciences, University of Oxford, Oxford; director, Royal College of General Practitioners Research and Surveillance Centre, London.

**Keywords:** COVID-19, frailty, polypharmacy, remote consultation, workload

## Abstract

**Background:**

The coronavirus disease 2019 (COVID-19) pandemic has resulted in a rapid change in workload across healthcare systems. Factors related to this adaptation in UK primary care have not yet been examined.

**Aim:**

To assess the responsiveness and prioritisation of primary care consultation type for older adults during the COVID-19 pandemic.

**Design and setting:**

A cross-sectional database study examining consultations between 17 February and 10 May 2020 for patients aged ≥65 years, drawn from primary care practices within the Oxford Royal College of General Practitioners (RCGP) Research and Surveillance Centre (RSC) sentinel network, UK.

**Method:**

The authors reported the proportion of consultation type across five categories: clinical administration, electronic/video, face-to-face, telephone, and home visits. Temporal trends in telephone and face-to-face consultations were analysed by polypharmacy, frailty status, and socioeconomic group using incidence rate ratios (IRR).

**Results:**

Across 3 851 304 consultations, the population median age was 75 years (interquartile range [IQR] 70–82); and 46% (*n* = 82 926) of the cohort (*N* = 180 420) were male. The rate of telephone and electronic/video consultations more than doubled across the study period (106.0% and 102.8%, respectively). Face-to-face consultations fell by 64.6% and home visits by 62.6%. This predominantly occurred across week 11 (week commencing 9 March 2020), coinciding with national policy change. Polypharmacy and frailty were associated with a relative increase in consultations. The greatest relative increase was among people taking ≥10 medications compared with those taking none (face-to-face IRR 9.90, 95% CI = 9.55 to 10.26; telephone IRR 17.64, 95% CI = 16.89 to 18.41).

**Conclusion:**

Primary care has undergone an unprecedented in-pandemic reorganisation while retaining focus on patients with increased complexity.

## INTRODUCTION

The coronavirus disease 2019 (COVID-19) pandemic has necessitated a rapid adaptation of patterns of clinical work in primary care. In the UK, as of 5 March 2020, GPs are recommended to use telephone or video triage wherever possible to reduce the number of patients needing to attend for face-to-face appointments.^1^ The Secretary of State for Health and Social Care stated: *‘The NHS will take a digital-first approach to accessing primary care and outpatient appointments’*,^2^ in an attempt to reduce the total footfall into practices, protecting both staff and patients.^3^ The impact of the implementation of these recommended changes to practice have not yet been quantified.

There is known variability in how COVID-19 affects different groups of the population. Older people and those with complex needs are more likely to have the disease, as well as suffer adverse outcomes.^4^^,^^5^ In addition, there are disparities on the impact of COVID-19, with people in deprived socioeconomic groups more likely to be negatively impacted by social distancing measures and loss of income, as well as being at greater risk of infection.^6^^,^^7^

This study assessed trends in face-to-face and remote consultations in response to the COVID-19 pandemic using routine primary care data. The authors focused on the population aged ≥65 years using frailty and polypharmacy as surrogates for complexity, as well as socioeconomic status, to explore whether these patients were being prioritised.

## METHOD

### Study population

Computerised medical record (CMR) data from patients aged ≥65 years registered with general practices within the Oxford Royal College of General Practitioners (RCGP) Research and Surveillance Centre (RSC) sentinel network, from 17 February to 10 May 2020, were obtained. This study focused on people aged ≥65 years as increasing age is associated with higher mortality from COVID-19 and because the electronic frailty index (eFI) is only validated for this age group.^8^^,^^9^

### Design and setting

A cross-sectional analysis of primary care consultations for people aged ≥65 years within the RCGP RSC sentinel network was conducted to quantify the impact of the COVID-19 pandemic on primary care work patterns.

RCGP RSC is one of the longest established primary care sentinel networks in Europe, and collaborates with Public Health England (PHE) on communicable disease surveillance.^10^ Since 2017, RCGP RSC has integrated analysis of workload trends into its standard activities.

**Table:** How this fits in

Primary care in the UK has undergone rapid reorganisation in response to the COVID-19 pandemic. Factors related to this adaptation in UK primary care have not yet been examined. In this study, across 3 851 304 consultations for adults aged ≥65 years between 17 February and 10 May 2020, rates of telephone and electronic/video consultations more than doubled (106.0% and 102.8%, respectively), while home visits and face-to-face consultations fell by 62.6% and 64.6%, respectively. Despite the shift in practice to a majority remote model, patients with complex needs were still prioritised. This study also found a degree of heterogeneity in the adoption of remote consultation at the practice level. Factors related to this variation need further exploration to establish if there are barriers to implementation of remote consulting approaches at the practice level.

Consultations were grouped using three patient-level variables, namely prescription counts, frailty status, and socioeconomic group. The number of prescriptions for an individual were categorised as 0 (reference), 1–4, 5–9, and ≥10, based on current active prescriptions at the time of analysis. Polypharmacy was defined as ≥5 prescribed medications and severe polypharmacy as ≥10 medications.^11^ Frailty status was determined using eFI.^9^ The eFI tool allows routine primary care data to be used to identify frailty across practice populations. This tool stratifies the population into fit, mild, moderate, and severe frailty groups. Deprivation status was based on scores from the English Index of Multiple Deprivation (IMD) and organised into quintiles of deprivation score (quintile 1 — most deprived; quintile 5 — least deprived).^12^ Consultations were classified into five types:

- clinical administrative consultations (non-patient facing, for example, where the clinician or administrative staff member codes secondary care consultations, completes medication administration or files results);
- electronic/video consultations (principally video-consultations but also includes emails to and from patients);
- face-to-face consultations;
- telephone consultations; and
- home visits, where the clinician visits a patient in their own home.

The codes for these different consultation types are shown in Supplementary Table S1.

### Statistical analysis

The same surveillance population was at risk of consulting each week, apart from minor missing representations, de-registrations, and deaths; a summary description of the cohort from week 8 only is provided in this article. Descriptive statistics were used to report the crude counts and rates of consultation types by week (Supplementary Table S2). Regression modelling was used to explore the consultation rates (per person-week) adjusted for frailty category, prescription group, and IMD quintile.

### Multivariate analysis

Multilevel negative binomial models were used to model rates for face-to-face and telephone consultations by each of the three patient-level variables (frailty, polypharmacy, and IMD quintile). No evidence was found to reject the model in a goodness-of-fit test (*P* is approximately equal to 1 for both models). Neither was evidence found of zero-inflation, with close concordance between predicted and observed events in the negative binomial model. Incidence rate ratios (IRRs) and 95% confidence intervals (CIs) are reported as well as *P*-values on estimated coefficients. Statistical analysis was carried out using R (version 3.5.0).

## RESULTS

### Population characteristics

Data from 3 851 304 consultations were analysed across the study period. Table 1 outlines the population characteristics at baseline (week 8), which is a stable representation of the population across the study period. The median age of the cohort was 75 years (interquartile range [IQR] 70–82) and 46% of the cohort were male (*n* = 82 926). There was evidence of a statistically significant burden of pre-existing disease within the study population. Few were taking no medications (*n* = 3376, 1.9%) and well over one-third were prescribed ≥10 medications (*n* = 74 231, 41.1%). Less than one in six were in the severely frail category (*n* = 26 925, 14.9%), but over half were categorised as mild or moderately frail (*n* = 107 604, 59.6%). There were relatively few consultations among people in the most deprived IMD quintile (*n* = 20 291, 11.2%) compared with the least deprived quintile, who consulted the most (*n* = 45 576, 25.3%).

**Table 1.** Characteristics of the cohort at baseline (week 8)

**Characteristics**	***n* (%)^a^ (*N*= 180 420)**	
**Age, years, median (IQR)**	75	(70–82)

**Sex**		
Male	82 926	(46.0)

**Polypharmacy group**		
0	3376	(1.9)
1–4	36 458	(20.2)
5–9	66 355	(36.8)
≥10	74 231	(41.1)

**Frailty category**		
Fit	45 891	(25.4)
Mild	64 330	(35.7)
Moderate	43 274	(24.0)
Severe	26 925	(14.9)

**IMD quintile**		
1 (most deprived)	20 291	(11.2)
2	25 213	(14.0)
3	37 156	(20.6)
4	43 520	(24.1)
5 (least deprived)	45 576	(25.3)

**Missing**	8664	(4.8)

a Unless otherwise stated. IMD = Index of Multiple Deprivation.^12^ IQR = interquartile range.

### Trends in consultation type

**Figure 1.**
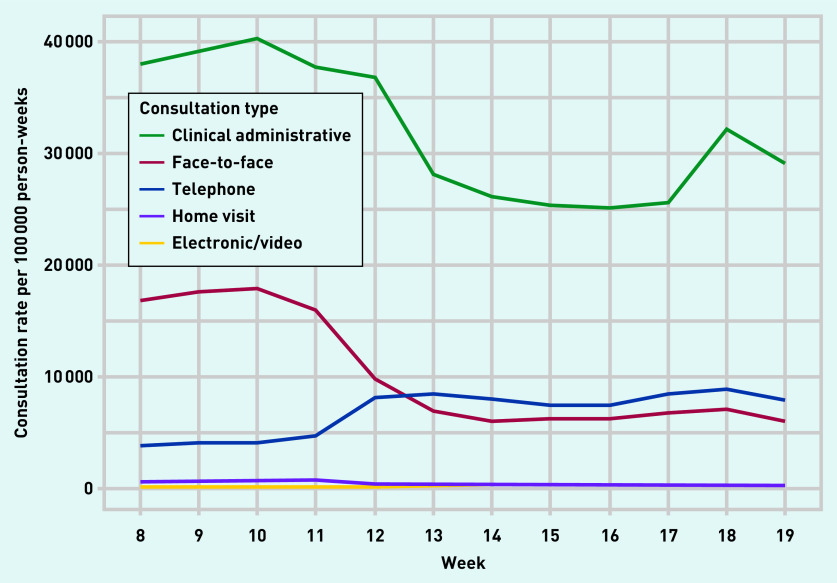
***Trend in consultation rates per 100 000 of the registered practice population by consultation type for weeks 8 to week 19, year 2020.***

A fall in face-to-face consultation rates by 64.6% (from 16 859 to 5966) from week 8 to week 19 of 2020 was observed; home visits by 62.6% (from 609 to 228); and clinical administrative appointments by 23.6% (from 38 038 to 29 049) (Figure 1 and Supplementary Table S2). Over the same period, telephone consults increased by 106.0% (from 3819 to 7866) and electronic/video consultations by 102.8% (from 106 to 215). Overall, the rate of consultations dropped by 27.1% (from 59 431 to 43 324).

Excluding clinical administrative appointments, the majority of which are not patient facing, telephone and electronic/video consultations made up 18.4% (17.9% teleconsultations, 0.5% electronic/video consults) of appointments in week 8, compared with 56.6% (55.1% teleconsultations, 1.5% electronic/video consultations) of appointments by week 14.

The biggest rate change in a single week occurred on week 11 (week commencing 9 March). In this single week, telephone consultations increased by 71.7% and electronic/video consultations by 26.6%, while there was a reduction of 54.0% for home visits, 38.7% in face-to-face consultations, and 2.3% in clinical administrative entries. Following this initial rapid adaptation, the changes in consultation type were sustained for the remaining study period. Week 11 coincided with the surge in community incidence of COVID-19, with weekly new cases rising from 2 per 100 000 at the start of week 11 to 79 per 100 000 by week 15 (Supplementary Figure S1). Clinical administrative consultations dropped by a further 23.7% between weeks 12 and 13, suggesting a lag in the impact of service change (Figure 1), which then recovered in week 18 with a 25.9% increase on the previous week.

### Changes in consultation patterns within patient groups

**Figure 2.**
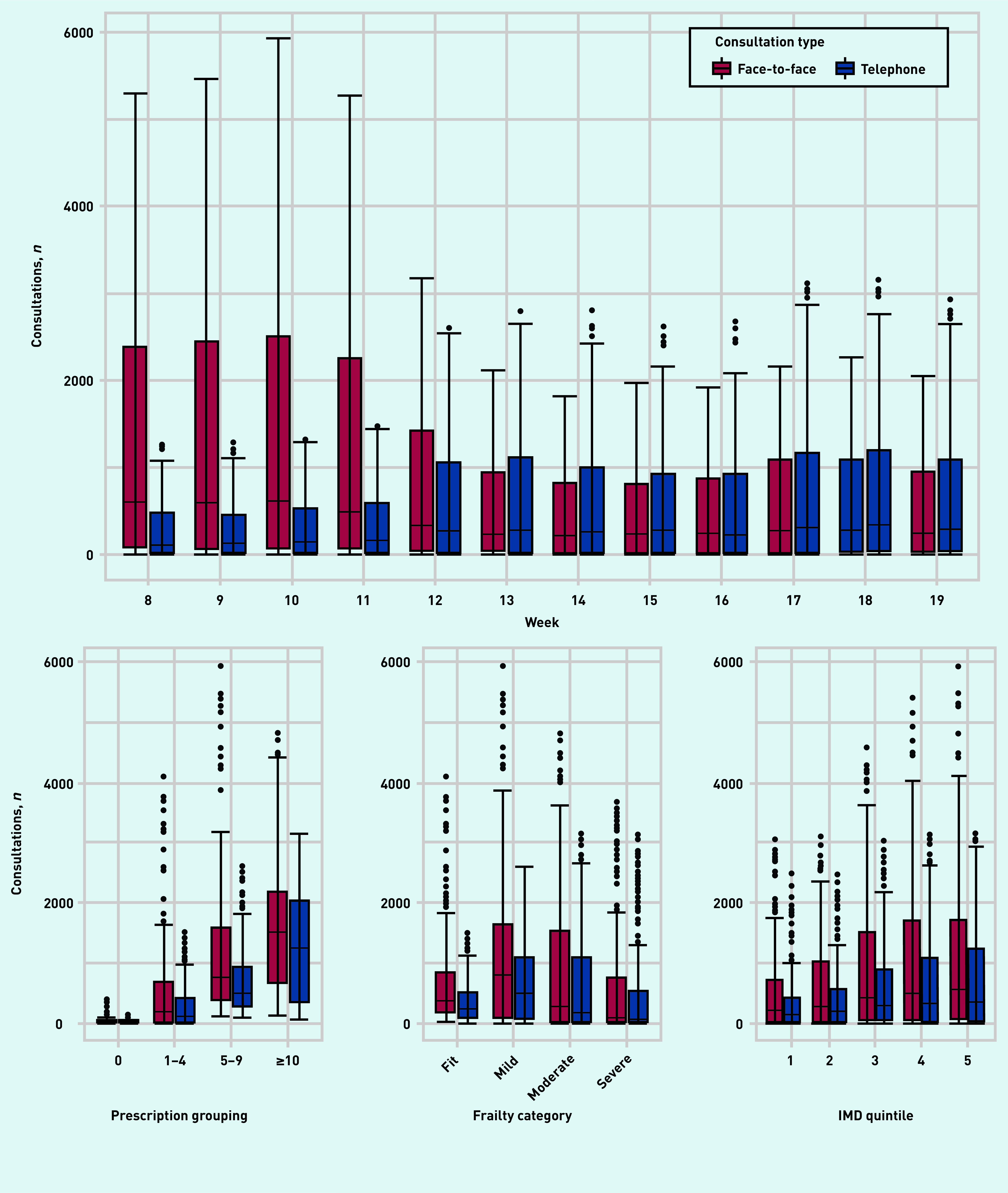
***Counts of telephone consultations and face-to-face consultations by week (of 2020) as well as by prescriptions group, frailty category, and IMD quintiles. IMD = Index of Multiple Deprivation.****^12^*

Changes in consultation rates varied between patient groups across the study period (Figure 2). For face-to-face consultations, the incidence rate ratio increased by >9 times for people taking ≥10 medications, compared with people on no medication (IRR 9.90, 95% CI = 9.55 to 10.26, Table 2). Increasing frailty was also associated with increased rates of face-to-face consultations compared with no frailty/fit (severe frailty versus fit IRR 1.64, 95% CI = 1.61 to 1.67). People in IMD quintile 1 (most deprived) saw a 5.2% increase in face-to-face consultations when compared with those in IMD quintile 5.

**Table 2.** Adjusted incidence rate ratios for face-to-face consultations

**Complexity**	**Adjusted IRR (95% CI)**		***P*-value**
**Prescription group (ref category: none)**			
1–4	3.3812	(3.2651 to 3.5014)	<0.001
5–9	5.5731	(5.3808 to 5.7724)	<0.001
≥10	9.8991	(9.5501 to 10.2608)	<0.001

**Frailty category (reference category: fit)**			
Mild	1.1794	(1.1627 to 1.1963)	<0.001
Moderate	1.3917	(1.3693 to 1.4145)	<0.001
Severe	1.6401	(1.6101 to 1.6706)	<0.001

**IMD quintile of deprivation (reference category: 3 – median quintile)**			
1 (most deprived)	0.9729	(0.9585 to 0.9875)	0.029
2	0.9851	(0.9711 to 0.9992)	<0.001
4	0.9426	(0.9281 to 0.9573)	<0.001
5 (least deprived)	0.9251	(0.9093 to 0.9412)	<0.001

**Week of 2020 (ref category week 8)**			
9	1.0391	(1.0207 to 1.0578)	<0.001
10	1.0615	(1.0428 to 1.0806)	<0.001
11	0.9436	(0.9265 to 0.9609)	<0.001
12	0.5712	(0.5594 to 0.5832)	<0.001
13	0.4007	(0.3913 to 0.4104)	<0.001
14	0.3401	(0.3319 to 0.3484)	<0.001
15	0.3556	(0.3466 to 0.3648)	<0.001
16	0.3652	(0.3560 to 0.3746)	<0.001
17	0.3968	(0.3873 to 0.4065)	<0.001
18	0.4159	(0.4064 to 0.4256)	<0.001
19	0.3497	(0.3417 to 0.3580)	<0.001

IMD = Index of Multiple Deprivation.^12^ IRR = incidence rate ratio.

The same pattern was seen in telephone consultation rates for polypharmacy and frailty. People prescribed ≥10 medications saw a 17-fold increase in telephone consultations compared with those on no medication (IRR 17.64, 95% CI = 16.89 to 18.41), with a relative increase in consultations for the frailest quintile. People in IMD quintile 1 (most deprived) saw a 7.7% increase in telephone consultations when compared with those in IMD quintile 5 (least deprived) (Table 3).

**Table 3.** Adjusted incidence rate ratios for telephone consultations

**Complexity**	**Adjusted IRR (95% CI)**		***P*-value**
**Prescription group (ref category: 0)**			
1–4	4.6410	4.4487 to 4.8415	<0.001
5–9	8.5436	8.1874 to 8.9153	<0.001
≥10	17.6362	16.8924 to 18.4128	<0.001

**Frailty category (reference category: fit)**			
Mild	1.2190	1.2019 to 1.2364	<0.001
Moderate	1.5105	1.4870 to 1.5344	<0.001
Severe	2.1117	2.0762 to 2.1479	<0.001

**IMD quintile of deprivation (reference category: 3 – median quintile)**			
1 (most deprived)	1.0114	0.9983 to 1.0246	0.0183
2	0.9847	0.9722 to 0.9974	0.0906
4	0.9594	0.9458 to 0.9732	<0.001
5 (least deprived)	0.9390	0.9242 to 0.9541	<0.001

**Week of 2020 (reference category: week 8)**			
9	1.0554	1.0247 to 1.0870	<0.001
10	1.0825	1.0514 to 1.1146	<0.001
11	1.2436	1.2094 to 1.2787	<0.001
12	2.1799	2.1258 to 2.2353	<0.001
13	2.2385	2.1829 to 2.2955	<0.001
14	2.0995	2.0467 to 2.1535	<0.001
15	1.9480	1.8986 to 1.9987	<0.001
16	1.9377	1.8883 to 1.9884	<0.001
17	2.2430	2.1876 to 2.2998	<0.001
18	2.3624	2.3044 to 2.4219	<0.001
19	2.0899	2.0384 to 2.1428	<0.001

IMD = Index of Multiple Deprivation.^12^ IRR = incidence rate ratio.

### Practice-level heterogeneity

There was a high degree of inter-practice variation in terms of adoption of remote consultation means, and considerable unexplained variation within the current model. Practices at the first quartile of the distribution of unobserved heterogeneity had 41% fewer telephone consultations and 53% fewer face-to-face consultations than expected from their observed characteristics, that is, prescribing group, frailty, IMD, and week, while those practices at the third quartile had 30% (telephone) and 35% (face-to-face) more than expected (Supplementary Table S3).

The indirectly standardised rates (Supplementary Figure S2) underline the evident variation in both teleconsultations and face-to-face consultation patterns across RCGP RSC practices.

**Figure 3.**
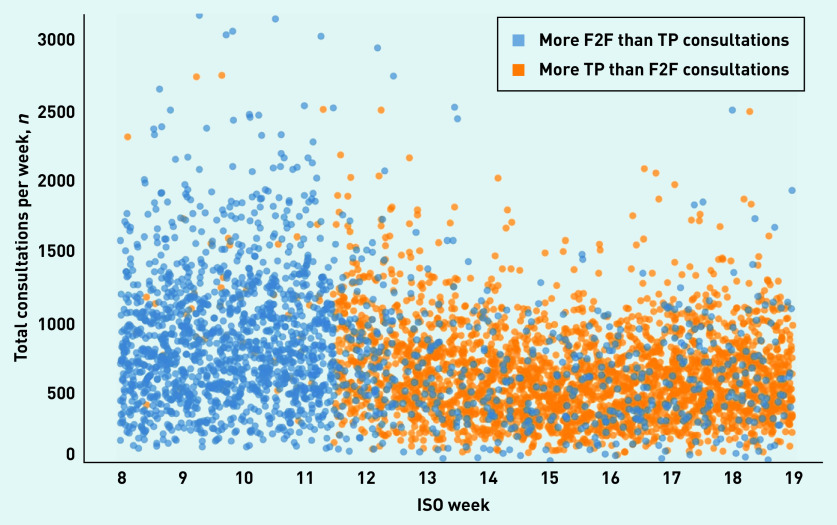
***Relative proportion of face-to-face (F2F) compared to telephone (TP) consultations at practices within the network. ISO = International Organization for Standardization.***

The rapid change from predominantly face-to-face to telephone consultations is again seen in Figure 3. However, at least 50 practices each week continued to offer the majority of appointments face-to-face throughout the study period (Supplementary Figure S3).

## DISCUSSION

### Summary

Data from the present study quantify the rapid primary care adaptations made in response to the COVID-19 pandemic. Telephone and electronic/video consultations more than doubled, alongside a 62.6% fall in home visits and a 64.6% fall in face-to-face consultations over the study period. This change predominantly occurred across week 11 (week commencing 9 March 2020), just 4 days after NHS England’s recommendation that GPs move to remote patterns of working to reduce face-to-face patient contacts.^1^ Remote consultation methods made up 18.4% of patient-facing appointments at baseline, compared with 56.6% by the end of the study period. People in the categories of highest frailty and polypharmacy saw the biggest relative increase in face-to-face and teleconsultations, suggesting the care of people with complex needs has been prioritised.

### Strengths and limitations

The presented data draw on a large, nationally representative, sentinel network of primary care practices. The authors suspect close to all clinical consultations were captured within the study design, though very occasionally healthcare professionals may have omitted to make an entry in the patient’s CMR.

Categorisation of consultation type relies on how this has been recorded within the original dataset. This may lead to inaccuracies, for example, where a clinician changes between type but does not record the switch. Length of consultation is not captured in these data, so fewer overall numbers of consultations do not necessarily equate to less work.

All analyses of consultation rates reflect activity rather than demand, and the number of consultations conducted is constrained by the number of appointments available. It is not possible to ascertain who initiated the consultation from these data, nor how many consultations individual patients had during this period. In the context of COVID-19 some practices or individual GPs may have proactively called patients they felt were most frail, or these patients may have been contacting their practice with greater frequency because they were more unwell. The impact of public health messages around the heightened risk of COVID-19 in vulnerable groups of patients is also likely to explain some of this effect, but this is not possible to disaggregate.

The present analysis does not capture changes in practice among younger populations, making it an incomplete analysis of overall consultation patterns. Although older patients contact GPs most frequently, it is likely the trends seen in this analysis are applicable to younger age groups. The analysis does not account for new initiatives within the healthcare system that may have reduced the number of consultations in primary care, such as the COVID Clinical Assessment Service (CCAS), established to support NHS 111 with remote clinical triaging.

Coding of frailty predominantly uses automated tools, such as the eFI, but these codes do not always accurately capture a clinician’s impression of the patient’s level of frailty. Indications for treatment and other comorbidities were not captured in the authors’ analysis and may be potential confounders for the reported associations.

### Comparison with existing literature

The observed fall in total consultations is in contrast to the long-term rise in workload and consultation rates seen in UK primary care.^13^ However, there has been a trend towards increasing teleconsultations over time, with a threefold increase between 1995 and 2008, albeit reaching only 12% of total consultations.^14^ A second study reported that GP telephone consultation rates doubled between 2007 and 2014, but 90% of all consultations remained face-to-face.^13^ In contrast, by week 19 in the presented study, 55.1% of patient-facing consultations were conducted by telephone and just 41.8% were face-to-face. To the authors’ knowledge, this is the first time that the majority of consultations in UK primary care have been conducted remotely.

There was a degree of inter-practice variation in the rates of both telephone and face-to-face consultations at weeks 8 and 19, indicating that some practices may have adapted to the digital-first approach more readily than others. Previous work on ‘telephone-first’ approaches in primary care demonstrate a large degree of inter-practice variation on overall workload, with a trend towards increased consultation time.^15^^,^^16^ A remote-triage approach may be more effective in data-driven practices and less likely to prove successful in practices where workload capacity struggled to meet demand at the outset.^15^ While the present observational study was conducted in entirely different circumstances, it provides a potential explanation for the heterogeneity observed.

Previous studies have reported higher consultation rates across all contact types for certain patient groups, such as older compared with younger patients, and those in the most deprived IMD quintile.^13^^,^^14^^,^^17^ In contrast, the present results suggest a trend towards increases in consultations for people in lower socioeconomic quintiles. However, the authors did not analyse comorbidities, such as diabetes and heart disease, or ethnicity, which have been reported as contributing factors to health disparities in COVID-19 and may explain the present findings.^7^^,^^18^^,^^19^

### Implications for research and practice

The comparative increase in consultation rates for people in the most deprived compared with least deprived socioeconomic groups is reassuring at face value, although more detailed investigation of relative covariates is warranted. Socioeconomic deprivation has been strongly linked to increased risk of death from COVID-19. For example, a recent UK analysis of primary care data reported the fully adjusted hazard ratio for in-hospital COVID-19-related mortality was 1.75 (95%, CI = 1.60 to 1.91) in the most deprived compared with least deprived IMD quintile.^20^ Further research is needed to understand what patient-, practice-, and public health-level factors influence patterns of consulting between different demographic groups to try to ensure the NHS addresses the ‘inverse care law’, where those most in need of help are least likely to get it.

There are opportunities to use the recent rapid changes in primary care implemented in response to COVID-19 as a platform for sustained changes in working patterns. Moving towards more remote consultations offers potential improvements in patient access and appointment flexibility. However, it also risks presenting an additional barrier to accessing care for people who are most vulnerable, such as those with limited access to the internet, smartphones, or other technology. Further evidence is needed to know whether the content of remote consultations is equivalent to face-to-face consulting, such as proportion that includes an opportunistic disease prevention or health promotion element. Future studies are needed to determine the impact of consultation type on length of appointments, overall workload, quality of care within the consultation, and patients’ experience of remote consultations, including possible barriers to accessing care.
